# Impact of strict IGF1 control on quality-of-life scores in patients with acromegaly

**DOI:** 10.3389/fendo.2025.1516899

**Published:** 2025-01-31

**Authors:** Chaitanya Gandhi, Marie-Claire Denis, Daniel Holmes, Juan Rivera, Stan van Uum, Shereen Ezzat, Constance Chik

**Affiliations:** ^1^ Department of Medicine, University of Alberta, Edmonton, AB, Canada; ^2^ Department of Medicine, Centre Hospitalier de l’Université Laval, Quebec City, QC, Canada; ^3^ Department of Pathology and Laboratory Medicine, St Paul’s Hospital, Vancouver, BC, Canada; ^4^ Department of Medicine, McGill University, Montreal, QC, Canada; ^5^ Department of Medicine, Western University, London, ON, Canada; ^6^ Department of Medicine, Princess Margaret Cancer Centre, Toronto, ON, Canada

**Keywords:** acromegaly, quality-of-life, pegvisomant, insulin-like growth factor 1, clinical trial

## Abstract

**Objective:**

Examine, in a real-world setting, whether strict normalization of modestly elevated insulin-like growth factor 1 (IGF1) results in clinical and health-related quality of life benefits in patients with acromegaly using an open-label, non-randomized, 6-month prospective interventional study.

**Methods:**

In patients with acromegaly and modest IGF1 elevation, strict IGF1 control was achieved by addition or dose escalation of pegvisomant. Clinical and biochemical parameters were assessed at baseline, 1 and 3 months for pegvisomant dose titration, and at 6 months. The Patient-Assessed Acromegaly Symptom Questionnaire (PASQ), the Acromegaly Quality of Life questionnaire (AcroQoL) and the Acromegaly Disease Activity Tool (ACRODAT^®^) were completed at baseline and at 6 months.

**Results:**

Ten patients (8 males) with mean age of 50.7 years participated in the study. All patients had a macroadenoma and nine had prior transsphenoidal surgeries. At time of screening, six patients were on a somatostatin analog, two on pegvisomant, and two on pegvisomant and a somatostatin analog. After six months of dose escalation or the addition of pegvisomant, IGF1 decreased from 1.22 ± 0.14 to 0.87 ± 0.20 times the upper limit of normal (p=0.001). PASQ score decreased by 3.5 (p=0.02) and the ACRODAT^®^ overall status decreased by 50.5 (p=0.001); however, there was no difference in the AcroQoL score. Hemoglobin A1c and liver enzymes did not differ and repeat MRI of the sella at 6 months showed no change.

**Conclusions:**

In this pilot study, stricter control of modest IGF1 elevations led to symptomatic improvement as measured by the PASQ score. These findings prompt larger prospective trials.

## Introduction

Acromegaly is a rare, chronic, debilitating, and insidious disease usually caused by a growth hormone (GH)-secreting pituitary tumor, which leads to GH and insulin‐like growth factor 1 (IGF1) excess (1, 2). Treatment goals include increasing life expectancy, reducing symptoms and signs of disease, and improving patients’ health-related quality-of-life (HRQoL) (2, 3). Biochemical treatment target includes normalization of age‐ and gender‐adjusted IGF1; however, IGF1 levels can be challenging to normalize, and even when normalized do not always correlate with improved HRQoL, symptoms and signs, or decreased burden of comorbidities (4, 5).

Treatment options for acromegaly include surgery, radiation, and medical therapy. In Canada, medical therapy with somatostatin analogs (SSAs) is recommended as first line agents (6–8). However, less than 50% of patients achieve IGF1 normalization with SSA monotherapy (7, 9). Moreover, HRQoL, symptoms and signs, as well as the burden associated with comorbid conditions often linger despite SSA monotherapy (9).

In comparison, pegvisomant (PEGV), a GH receptor antagonist, has been shown to normalize IGF1 levels in up to 90% of patients in whom SSA therapy was ineffective (10, 11). PEGV is also indicated if there is inadequate response to or inability to tolerate surgery, radiation therapy, and other medical therapies (10–12). In addition to normalizing IGF1 levels, PEGV has been demonstrated to have a positive impact on patients’ HRQoL, symptoms and signs of acromegaly and comorbidities (especially dysglycemia) (12–15). Two studies where PEGV was used in combination with SSAs also showed sustained improvement in HRQoL scores (5, 16). The very definition of patient control and IGF1 target varies from study to study, in which IGF1 levels of 1.0 to 1.3x upper limit of normal (ULN) can be found, with stricter control in more recent studies (16–20).

Together these studies suggest that normalization of IGF1 levels in patients insensitive to SSAs (i.e., IGF1 levels > 1.3x ULN) may benefit from symptomatic HRQoL improvements. Therefore, the objective of our pilot study was to determine if patients with acromegaly and modest IGF1 elevations (IGF1 levels 1 to 1.3x ULN) would benefit from stricter normalization of IGF1 with addition or dose escalation of PEGV therapy.

## Methods

### Patients

**Figure 1:**
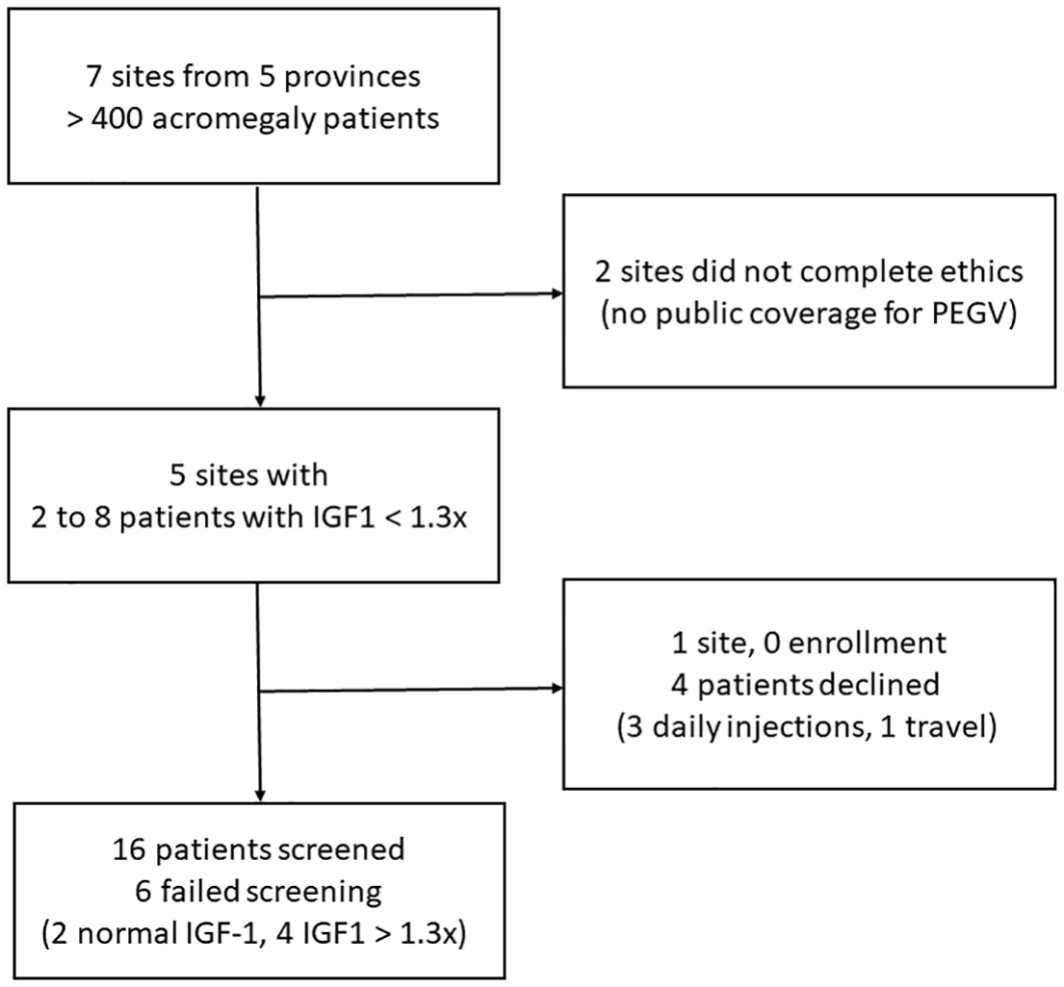
A flow chart illustrating the recruitment process; IGF1, insulin-like growth factor 1; PEGV, pegvisomant.

This trial included adult patients with confirmed acromegaly who had been on SSA, PEGV, or their combination but remain with modest elevation in serum IGF1 levels adjusted for age (1.0x ULN < [serum IGF1] < 1.3x ULN). **Figure 1** is a flow chart illustrating the recruitment process. Due to recruitment issues and variabilities with IGF1 measurements, 2 patients with IGF1 between 1.3 to 1.5x ULN were also included in the study. Patients were excluded from our trial if they met any of the following key criteria: visual field loss, pituitary tumors compressing or ≤ 3 mm from optic chiasm, cranial nerve palsies requiring urgent tumor decompressive surgery, pre-existing liver disease (defined as alanine aminotransferase or aspartate aminotransferase > 3x ULN), pituitary surgery or radiation therapy within one year prior to screening visit, allergy to PEGV, pregnancy, substance-use disorder, or inability to inject PEGV.

### Study design

This trial was an open‐label, non‐randomized, single arm, variable dose study of PEGV conducted in a real-world multicenter setting (clinicaltrials.gov, NCT02952885). The primary purpose of the study was to measure the changes in biochemical, clinical and HRQoL parameters before and after the administration of PEGV as monotherapy, or as adjunct therapy in patients who were partially responsive to SSA monotherapy, and who remained on SSA alone or have started combination therapy of SSA and PEGV, but whose IGF1 remained elevated at 1 to 1.3x ULN. Efficacy data were assessed by means of central laboratory measurements of IGF1, patient reported HRQoL, acromegaly symptoms and signs, and assessment of comorbidities. Safety data, including treatment‐related adverse events (AEs), subject discontinuations due to AEs, and withdrawals were collected. The study protocol was approved by the individual center’s Health Sciences Research Ethics Board. All participants gave written informed consent prior to the study. The study was conducted in accordance with the ICH‐GCP guidelines and the principles of the Declaration of Helsinki.

### Interventions

Study medications were prescribed as per clinical practice, with PEGV being initiated or optimally dosed at the initial visit (Month 0). PEGV was dosed at 5 to 10 mg daily if used as combination therapy with SSA or 20 mg daily if used as monotherapy. Dosing of PEGV was adjusted as per clinical judgment to meet normalization of IGF1 levels (< 1.0x ULN) in increments of 5 to 10 mg daily with a predetermined maximum dose of 40 mg daily. Dose adjustments of PEGV occurred at screening (Month 0), if already on PEGV, Month 1, and Month 3. In the event of a reduction in IGF1 below the lower limit of normal, the dose of PEGV was decreased by 5 to 10 mg daily. IGF1 measurements were completed in the central laboratory at the St. Paul’s Hospital, Vancouver, British Columbia, Canada. The first six patients’ samples were processed using the IDS-iSYS platform (Immunodiagnostic System Ltd, Boldon, UK) and the method was then transitioned to the Roche Elecsys^®^ (Roche Diagnostics, Laval, Quebec, Canada). The transition had no impact on our study results (21) and these two analytic methods have the best agreement among eight immunoassays in a recent study (22).

### Outcomes

The primary outcome of this study was HRQoL improvement at 6 months as assessed by AcroQoL (23), acromegaly symptoms and signs as assessed by the PASQ scale (5), and overall acromegaly status as determined by the ACRODAT^®^ software (24), compared to baseline. Secondary outcomes included change in serum IGF1, glycosylated hemoglobin, lipid profile and blood pressure at 6 months compared with baseline.

Safety outcomes were monitored by assessment at each visit of serum electrolytes, renal function, liver function (months 1, 3 and 6). A magnetic resonance imaging of the sella was obtained at month 6 to assess for possible tumor growth.

### Statistical analysis

Demographic and clinical data were summarized as means with standard deviations for continuous variables, and by absolute numbers and percentages for categorical variables. Changes in AcroQoL, PASQ, ACRODAT^®^ scores, IGF1, hemoglobin A1c, lipid panel, and blood pressure were analyzed by a paired t-test, the Wilcoxon signed-rank test, correlation and regression or chi-square as appropriate. The preliminary statistical analysis was performed by the Applied Health Research Centre in St. Michael’s Hospital, Toronto, Ontario, Canada. A P-value <0.05 was considered statistically significant.

## Results

### Patient characteristics

Ten patients (8 male and 2 female) were included in this study. **Table 1** demonstrates the baseline characteristics of patients at diagnosis of acromegaly and at the time of enrollment in the study. The mean enrollment age was 50.7 years (range 36 to 64 years) and the mean disease duration was 6.8 years (range 2.3 to 20.6 years). Of 10 patients, seven were Caucasian, one African, one Indigenous, and one of Middle Eastern descent. Acromegaly-related comorbidities included colonic polyps (n=5), hypertension, dyslipidemia, and sleep apnea (n=4 each), diabetes mellitus, osteoarthritis, and goiter (n=3 each), and cardiac disease, cancer, and psychological disorder (n=1 each).

**Table 1:** Baseline demographic and clinical characteristics of the 10 patients.

Parameters	Value (Mean ± SEM)
Age (years)	50.7 ± 11.3
Duration of illness (years)	6.80 ± 6.41
Height (cm)	176.4 ± 12.5
Weight (kg)	89.7 ± 19.7
Body mass index (kg/m^2^)	28.7 ± 6.0
Heart rate (bpm)	69.3 ± 15.4
Blood pressure – systolic (mmHg)	131.2 ± 16.1
Blood pressure – diastolic (mmHg)	81.2 ± 13.2
IGF1 central labs (μg/L)	258.5 ± 59.2
IGF1 local labs (μg/L)	271.3 ± 81.6
Fasting growth hormone (μg/L)	5.54 ± 6.49

IGF1, insulin-like growth factor 1.

All patients had a macroadenoma at baseline, including seven with radiologically invasive tumors and two with suprasellar extension. Transsphenoidal surgery was the initial management choice for nine patients and one patient also had radiation therapy. All ten patients had trials of SSA (eight with Sandostatin long-acting repeatable and two with Lanreotide Autogel). Four patients had trials of SSA in combination with cabergoline which failed to normalize IGF1. SSA was switched to PEGV in two patients because of lack of efficacy. At the time of screening, six patients were on a stable dose of SSA, two patients were on PEGV, and two patients were on combination SSA and PEGV. Anterior hypopituitarism was present in seven patients.

**Figure 2:**
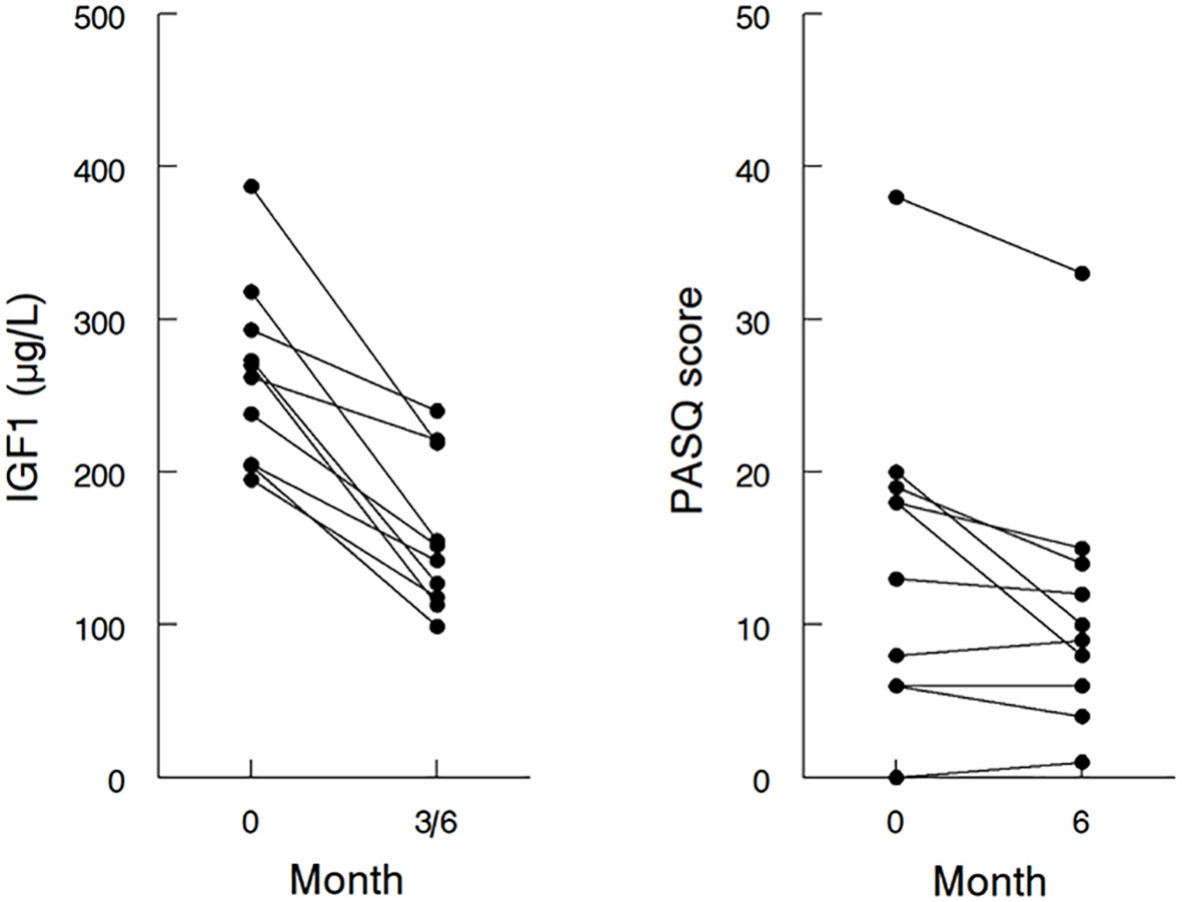
Effects of addition and/or dose escalation of pegvisomant on IGF1 and PASQ in individual patients. IGF1 at baseline and the lowest IGF1 attained at 3 or 6 months (left panel), and the sum of PASQ1 to PASQ6 at baseline and at 6 months (right panel) after the addition or dose escalation of pegvisomant; IGF1, insulin-like growth factor 1; PASQ, Patient-Assessed Acromegaly Symptom Questionnaire.

**Table 1** shows the anthropometric parameters of the participants. The mean hemoglobin A1c was 6.6% with 3 patients meeting the criteria for a diabetes mellitus diagnosis and two had uncontrolled diabetes. Baseline lipid panel is listed in **Table 2**. Mean serum IGF1 at baseline was 258.5 μg/L (1.22x ULN) with fasting GH of 5.54 μg/L (**Table 1**). **Figure 2** (left panel) shows the Individual baseline serum IGF1.

**Table 2:** IGF1, fasting glucose, hemoglobin A1c, lipid profile, liver function, kidney function and electrolytes at baseline (Month 0) and at the end of the trial (Month 6).

Parameters	Month 0	Month 6	P value
Biochemical investigations			
IGF1 central labs (age-adjusted ULN)	1.15 ± 0.12	0.84 ± 0.28	0.002*
IGF1 local labs (age-adjusted ULN)	1.22 ± 0.14	0.87 ± 0.20	<0.001*
Fasting glucose (mmol/L)	6.30 ± 1.57	6.32 ± 1.96	0.91
Hemoglobin A1c (%)	6.56 ± 1.20	6.34 ± 1.04	0.16
Total cholesterol (mmol/L)	4.21 ± 0.86	4.08 ± 0.88	0.46
High-density Lipoprotein (mmol/L)	1.29 ± 0.43	1.31 ± 0.36	0.66
Low-density Lipoprotein (mmol/L)	2.26 ± 0.94	2.19 ± 0.94	0.70
Triglycerides (mmol/L)	1.61 ± 1.23	1.28 ± 0.66	0.25
Alanine aminotransferase (U/L)	30.3 ± 17.2	25.5 ± 13.0	0.09
Aspartate aminotransferase (U/L)	25.9 ± 10.3	24.7 ± 10.4	0.78
Gamma-glutamyl transpeptidase (U/L)	22.9 ± 18.5	27.2 ± 17.6	0.69
Total bilirubin (µmol/L)	10.4 ± 3.9	10.6 ± 4.6	0.88
Alkaline phosphatase (U/L)	65.5 ± 26.3	64.9 ± 25.2	0.95
Creatinine (µmol/L)	81.6 ± 17.0	83.5 ± 13.7	0.78
Blood urea nitrogen (mmol/L)	5.10 ± 1.25	5.54 ± 1.87	0.54
Sodium (mmol/L)	139.7 ± 3.4	139.3 ± 2.4	0.53
Potassium (mmol/L)	4.22 ± 0.36	4.23 ± 0.38	0.85
Chloride (mmol/L)	103.9 ± 2.7	104.3 ± 2.8	0.76
Bicarbonate (mmol/L)	27.3 ± 2.7	26.6 ± 3.2	0.38

*statistically significant; IGF1, insulin-like growth factor 1.

### Outcomes

At the end of six months, the mean daily dose of PEGV had increased from 7.5 ± 12.3 mg (range 0 to 30 mg daily) to 17.6 ± 13.7 mg (range 5 to 40 mg daily). The addition or dose-optimization of PEGV resulted in a lowering of the mean IGF1 from 1.22 to 0.87x ULN (p=0.001) (**Table 1**). The maximal IGF1 lowering of each individual patient is shown in **Figure 2** (left panel). One patient did not have normalization of IGF1 during the study because he was reluctant to further increase the dose of PEGV during the COVID-19 pandemic. In 3 patients, the maximal lowering of IGF1 levels was observed at 3 months, with higher IGF1 levels at 6 months despite unchanged dose of medications.

IGF1 lowering was accompanied by a slight increase in the mean AcroQoL score by 2.7 (**Table 3**); however this difference was not statistically significant (p=0.11). The pre- and post-treatment AcroQoL global score of individual patients is shown in **Supplementary Figure S1**. In comparison, there was a statistically significant change in the sum of PASQ1 to 6 score with a mean difference of -3.5 (p=0.02) (**Table 3**). The pre- and post-treatment value of the individual patient is shown in **Figure 2** (right panel). Whereas patients with initial PASQ scores > 15 showed symptomatic improvement with IGF-1 normalization, those with relatively less impairment and baseline PASQ scores <15 showed minimal change (6.6 ± 3.2 vs 0.2 ± 1.1, P=0.003).

**Table 3:** AcroQoL, total PASQ score and ACRODAT^®^ overall status at baseline (Month 0) and at the end of the trial (Month 6).

Outcome	Month 0	Month 6	P value
AcroQoL global score	63.8 ± 24.0	66.5 ± 22.6	0.11
AcroQoL normalized subscale scores			
Physical subscale	60.9 ± 25.6	62.8 ± 24.6	0.33
Psychological/ Appearance subscale	58.9 ± 26.6	63.6 ± 24.0	0.15
Psychological/Personal relations subscale	71.8 ± 23.2	73.6 ± 25.0	0.87
Sum of PASQ1 to PASQ6	14.6 ± 10.6	11.1 ± 8.88	0.02*
PASQ1 - headache	1.8 ± 2.1	1.8 ± 2.6	1.00
PASQ2 - excessive sweating	2.1 ± 2.7	1.4 ± 2.2	0.31
PASQ3 - joint pain	3.2 ± 2.7	2.1 ± 2.3	0.06
PASQ4 - fatigue	2.9 ± 2.8	3.1 ± 2.5	0.51
PASQ5 - soft tissue swelling	2.3 ± 2.2	1.6 ± 2.0	0.09
PASQ6 - numbness or tingling	2.3 ± 2.6	1.1 ± 1.4	0.04*
PASQ7 - overall status	2.7 ± 2.5	2.3 ± 2.4	0.44
ACRODAT^®^ overall status	84.3 ± 27.6	33.8 ± 17.1	0.001*
IGF1 score	2.4 ± 0.5	1.4 ± 0.5	<0.001*
Total ACRODAT score not including IGF1	7.1 ± 1.8	6.4 ± 1.8	0.02*

*statistically significant; ACRODAT, Acromegaly Disease Activity Tool; AcroQoL, Acromegaly Quality of Life; IGF1, insulin-like growth factor 1; PASQ, Patient-Assessed Acromegaly Symptom Questionnaire.

Among the six PASQ questions addressing the different symptoms, improvement was observed in numbness or tingling (p=0.04), and a trend towards improvement was seen for joint pain and soft tissue swelling (p=0.06 and 0.09, respectively). There was no correlation between the changes in the sum of PASQ1-6 score and the AcroQoL global score (r = -0.19, P=0.60) and no specific variable (hypopituitarism, sleep apnea, diabetes, hypertension, osteoarthritis) was identified that could distinguish those with an improvement ≥ 5 in their PASQ1-6 score (data not shown).

On the ACRODAT^®^ scale, an improvement was observed in the overall status score which was reduced from 84.3 to 33.8 (p=0.001) (**Table 3**). We also noted a difference in the summation of the scores for tumor status, comorbidities, symptoms, and HRQoL impairment (-0.7, p=0.02). There was no significant change in blood pressure (131.2 ± 16.1/81.2 ± 13.2 mmHg at baseline and 128.0 ± 15.2/81.2 ± 12.8 mmHg at 6 months), hemoglobin A1c, and lipid panel after 6 months of addition or intensification of PEGV therapy (**Table 2**).

### Safety

Three moderate AEs (fall, unstable angina, and right sub-mandibular abscess) and 11 mild AEs (chest pain, conjunctivitis, hip pain, headache, congestion, fatigue, hypertension, knee pain, left finger joint pain, injection site redness and herpetic whitlow finger) were reported in eight patients. All AEs were transient and had completely resolved by month 6. Importantly, liver enzymes did not differ at the end of the study (**Table 2**) and there was no change in the pituitary lesion in the repeat MRI at 6 months.

## Discussion

Normalization of IGF1 and reduction of GH levels (< 2.5 μg/L) reverses the excess mortality seen in untreated acromegaly to that of the general population (3, 4). Hence, conventional acromegaly treatment outcomes have focused on biochemical and radiological criteria, such as improvements in IGF1, GH, and pituitary tumor size (9). However, the current acromegaly treatment paradigm also considers additional patient-centred outcomes such as HRQoL to measure status of ongoing comorbidities and residual symptoms and signs (e.g. diaphoresis) (4, 5). Importantly, serum IGF1 and GH have been shown to be incomplete predictors of HRQoL in acromegaly. Indeed, surveys of acromegaly patients have highlighted the significance of symptomatic parameters in addition to conventional biochemical tools when assessing disease control of acromegaly (5, 19, 25, 26).

To further explore the relationship between IGF1 normalization and HRQoL improvement, in this study, we assess in a real-world setting whether normalization of modestly elevated serum IGF1 would significantly impact clinical parameters and HRQoL in patients with acromegaly. Our results shows that PEGV at a mean dose of 17 mg daily was effective in reducing IGF1 from 1.22 to 0.87x ULN, with normalization of IGF1 in nine of ten patients. This is similar to results in previous studies where PEGV was effective in normalizing IGF1 in up to 90% of patients (12–14).

In our small cohort of participants, although strict IGF1 control, i. e. normalization, compared with modestly elevated IGF1 (1.0 to 1.5x ULN), did not improve the AcroQoL score, the sum of PASQ1-6 score and the ACRODAT^®^ overall status showed measurable improvements. Previous studies have also shown poor correlation between IGF1 levels and the AcroQoL score. There has been speculations that AcroQoL may not be sensitive enough to detect small differences in HRQoL especially in patients with modest IGF1 elevation (4, 18). Other studies have shown that the addition of PEGV therapy alone results in improved AcroQoL scores, especially on the physical subscale (5, 17). In a recent study, based on real-world experience, IGF1 control with PEGV therapy had no effects on AcroQoL scores; however long term use resulted in small improvements in PASQ scores (27). In our study, the small sample size and short duration of follow-up may have been insufficient to detect a change in the AcroQoL score. Alternatively, given AcroQoL’s comparatively greater focus on psychosocial factors compared to PASQ/ACRODAT^®^, a significant difference may not have been apparent (23).

Whereas the PASQ score focuses on symptoms and signs related to acromegaly, the ACRODAT^®^ overall status emphasizes a combination of paraclinical (IGF1 levels, tumor size) and clinical factors (comorbidities, symptoms, and HRQoL) (5, 24). Improvement in the sum of PASQ1-6 score and the ACRODAT^®^ overall status in our study (**Table 3**) suggest that the main effect of strict IGF1 control is related to improved symptom control. In the case of the ACRODAT^®^ overall status, even after exclusion of IGF1, the summation of the scores for symptoms and HRQoL impairment remained significant. Taken together, given the lack of significant improvement in AcroQoL, in addition to lowering of IGF1, another driver of improvement in ACRODAT^®^ is improvement in symptom control.

A possible basis for PEGV-induced improvement of QoL symptoms may be related to its mechanism of action (28). The “extra-hepatic” acromegaly hypothesis, which has been put forth by Neggers and colleagues, proposes that SSA effect reduces proportionally more hepatic IGF1 production compared with GH production, leading to disproportionately elevated peripheral GH responsible for perpetuating some acromegaly symptoms (29). The addition of PEGV antagonizes peripheral GH receptors, thereby diminishing GH activity throughout the body (15). Indeed, even when circulating IGF1 is within age-adjusted normal range with SSA monotherapy, addition of PEGV has been shown to improve PASQ (5). In contrast, although HRQoL improved with SSA monotherapy, changes in PASQ scores were independent of biochemical control (30).

In regard to important cardiovascular risk factors associated with acromegaly, in our small series, there were no differences in hemoglobin A1c, lipid panel, and blood pressure after six months of treatment. This in part reflected reasonable control of these parameters at baseline (hemoglobin A1c 6.6%, low-density lipoprotein 2.26 mmol/L, and blood pressure 131/82 mmHg) and management options (lifestyle or pharmacotherapy) employed to treat these comorbidities. Among the three patients with diabetes, one with uncontrolled diabetes had improved hemoglobin A1c with intensification of insulin therapy. Previous studies have demonstrated improved glycemic control (12, 13) with PEGV treatment; however glycemic control may not necessarily improve with combination therapy (31, 32). In our study, eight of ten patients were on combination therapy.

Eight patients reported 14 AEs, none were severe and all were transient with eight possibly related to the use of PEGV. Previous studies have demonstrated transient elevation in liver enzymes with PEGV, which usually resolved after therapy cessation and seemed to occur more frequently in patients with diabetes mellitus and when used in combination with SSA (14, 17). Even though eight of ten patients were on combination therapy, elevated liver enzymes were not seen in our study; however, the duration of the study was only six months.

Limitations of our study include relatively small sample size, largely due to difficulty obtaining medication coverage for patients with mild elevations of IGF1 in some Canadian provinces, and the study being conducted during the COVID-19 pandemic. Other limitations include short duration of study, lack of a control group due to an open-label design, and heterogeneity of patient population. Also, seven patients had hypopituitarism and one patient had prior radiation therapy, which may be confounding factors as they are known to contribute to impaired HRQoL. Moreover, given the well recognized variabilities of the IGF1 measurements, in particular in patients who are on SSA (33, 34), optimal PEGV dosing was not possible in a subset of our patients. There also was a gender imbalance in our study with two female and eight male participants, whereas in an epidemiological review of population studies, there is usually an equal distribution of acromegaly prevalence between males and females (35, 36). However, two previous studies have indicated higher prevalence rates in men than in women, including a male-to-female ratio of 1:1.8 in an older study (37, 38). The applicability of our study to the general population in countries where the public payer does not cover PEGV may also be difficult due to its expensive cost. Other options to normalize IGF1 such as the addition of cabergoline to SSA was only used in four of ten patients prior to the study (39).

In spite of the small number of patients participating in this trial, strict control in acromegaly patients with modest IGF1 elevations by PEGV initiation or dose escalation was accompanied by clinical improvements detected by the PASQ score. Whereas patients with worse initial PASQ scores showed improvement with IGF-1 normalization, those with relatively preserved baseline PASQ scores showed minimal change. This observation implies that our findings may not be applicable to all acromegaly patients. Instead, the results suggest that stringent IGF-1 control might be particularly beneficial for patient subgroups with more impaired HRQoL and a larger scale clinical trial will need to be completed to confirm this finding.

## Data Availability

The original contributions presented in the study are included in the article/**Supplementary Material**. Further inquiries can be directed to the corresponding author.

## Supplementary material

Supplementary Figure 1 Effect of addition and/or dose escalation of pegvisomant on the AcroQoL global score in individual patients at baseline and at 6 months. The two patients with the biggest improvement in the sum of PASQ1 to PASQ6 score were depicted with a blue line; PASQ, Patient-Assessed Acromegaly Symptom Questionnaire.
