# Supplementary figures and images for: Impact of strict IGF1 control on quality-of-life scores in patients with acromegaly
Source: Front Endocrinol (Lausanne). 2025 Jan 31;16:1516899. doi: 10.3389/fendo.2025.1516899 (PMC11825318; doi:10.3389/fendo.2025.1516899)

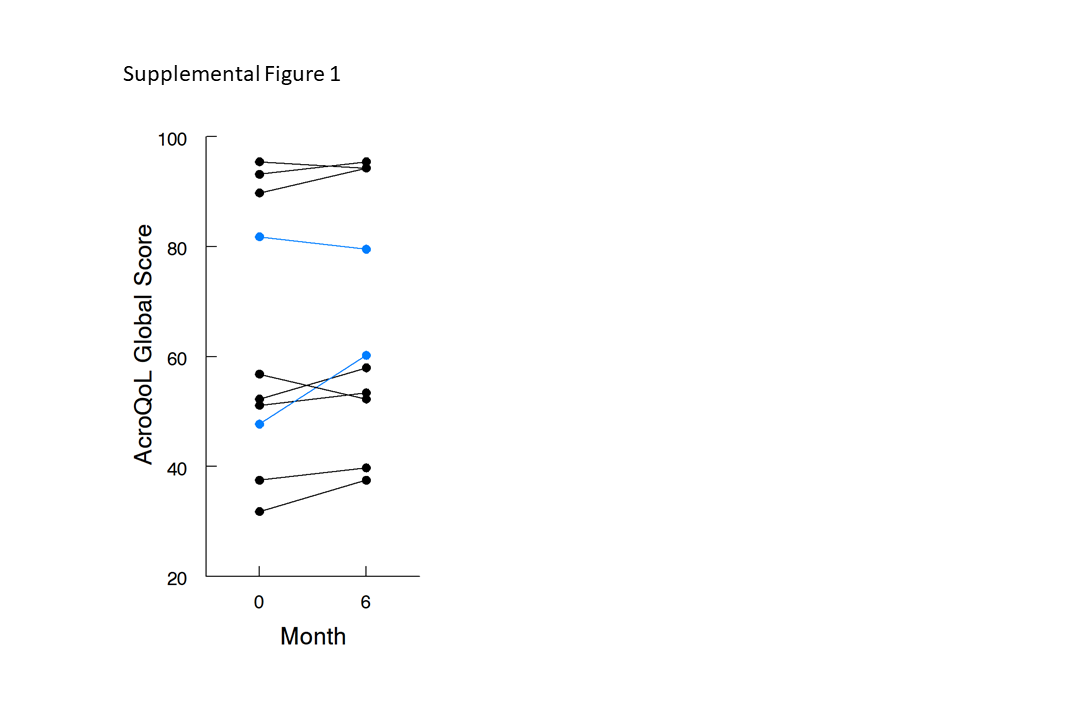

Supplement: Supplementary Figure 1 — Effect of addition and/or dose escalation of pegvisomant on the AcroQoL global score in individual patients at baseline and at 6 months. The two patients with the biggest improvement in the sum of PASQ1 to PASQ6 score were depicted with a blue line; PASQ, Patient-Assessed Acromegaly Symptom Questionnaire. [file Image1.tif]
